# Diagnostic Efficacy of Lsa63 Antigen for Human Leptospirosis

**DOI:** 10.5812/ircmj.14753

**Published:** 2014-03-05

**Authors:** Safar Ali Alizadeh, Seyyed Saeed Eshraghi, Mohammad Reza Pourmand, Taghi Naserpour, Gholamreza Abdollahpour, Abbas Rahimiforoshani, Reza Najafipour

**Affiliations:** 1 Department of Pathobiology, School of Public Health, Tehran University of Medical Sciences, Tehran, IR Iran; 2 Department of Microbiology, Molecular Research Center, Qazvin University of Medical Sciences, Qazvin, IR Iran; 3 Faculty of Veterinary Medicine, University of Tehran, Tehran, IR Iran; 4 Department of Epidemiology and Biostatistics, School of Public Health, Tehran University of Medical Sciences, Tehran, IR Iran; 5 Department of Biochemistry, Genetics Research Center, Qazvin University of Medical Sciences, Qazvin, IR Iran

**Keywords:** Enzyme-Linked Immunosorbent Assay, *Leptospira*, leptospirosis, Lsa63

## Abstract

**Background:**

Timely diagnosis of leptospirosis is essential for early and effective treatment, for there are many differential diagnoses for it.. Leptospiral researchers have an increasing interest in developing new serological methods with recombinant antigens to improve the Leptospirosis diagnosis. Several serological tests have been developed for the proper diagnosis of leptospirosis.

**Objectives:**

To improve the previous works we developed an enzyme linked immunosorbent assay (ELISA) with novel recombinant leptospiral surface adhesion (Lsa63) protein to offer a new test.

**Materials and Methods:**

In an experimental study, Recombinant Lsa63 (rLsa63) was produced in *E*scherishia* coli* (E.coli) BL21 (DE3). By using rLsa63, we generated IgM and IgG ELISA. Performance of these tests was compared to microscopic agglutination golden test (MAT). Two hundred twenty human serum samples were obtained from individuals suspicious of leptospirosis who were referred to Guilan Province Central *Leptospira* Laboratory for definitive diagnosis. The sensitivity, specificity and other statistical indexes of Lsa63-ELISAs were also determined.

**Results:**

Among 220 serum samples, 30% (n = 65) had positive MAT responses, and also 38% (n = 84) and 40.9% (n = 90) showed positive reaction to IgG and IgM rLsa63-ELISA, respectively. The sensitivity, specificity and accuracy were 93.8%, 81.29 % and 85.0 for IgM-Lsa63- ELISA and 83.07, 80, 64 and 81.36 for IgG-Lsa63- ELISA, respectively.

**Conclusions:**

Our results demonstrated that the sensitivity and specificity of Lsa63-ELISAs are promising for the detection of *Leptospira* serovars.

## 1. Background

Leptospirosis is a common re-emerging and zoonosis infectious disease worldwide, caused by pathogenic spirochetes of the genus *Leptospira* (1, 2). Humans are infected after a skin or mucosal exposure to animal reservoir or materials like water, food and soil contaminated by animal’s urine (3, 4). The incidence of this disease is significantly higher in tropical and subtropical regions (5, 6). High risk groups are mostly rice farmers, veterinarians, slaughterhouse workers, butchers, fishermen, sewer maintenance workers, laboratory staff and employees of waste disposal facility (7, 8). Clinical manifestations of leptospirosis varies from mild to severe and symptoms include fever, myalgia, headache, malaise, intense jaundice and bleeding (9-11). The most severe forms of it include Weil’s disease, pulmonary hemorrhage syndrome and liver or renal failure may also develop and consequently lead to death (12-14).

Timely diagnosis of leptospirosis is essential for early and effective treatment. The major concern with leptospirosis diagnosis are its similarities to other diseases, which makes it difficult to differentiate from other febrile conditions like influenza, hemorrhagic fevers, typhoid fever, rickettsiosis, aseptic meningitides, hepatitis and malaria (15, 16). Right now, techniques usually used in laboratories for diagnosis of leptospirosis are bacteriological cultures, microscopic agglutination test (MAT), serological tests (ELISAs, agglutination tests and IFA) and molecular tests (PCRs, Real time PCRs) (17, 18). Each of these methods has some limitations for specific and sensitive diagnosis of leptospirosis. The difficulty with the present tests had researchers to look for new methods. Usually the serological methods are prefered by researchers for developing new tests due to their availability and simple protocols. The new developing tests should be more specific, sensitive and cost effective for public laboratories. Their accurate specificity also needs to be improved. Consequently, many new serological tests have been developed to solve the existing problems with the diagnosis of leptospirosis. The aim of the present survey was to study leptospiral surface adhesion of 63 kDa (Lsa63) protein for establishing an ELISA to develop a new test for exploring the potential capability of Lsa63 for the detection of anti *Leptospira* spp. antibodies and evaluate it for serodiagnosing of leptospirosis. This protein is encoded by the gene LIC10314 (1650 bp) and expressed on the surface of pathogenic *Leptospira interrogans* serovar Lai, in both strains of *Leptospira borgpertensenii*. Monica L. Vieira introduced Lsa63 previously as a novel surface protein of pathogenic *Leptospira* spp. (13). Like other surface proteins in different bacteria, this protein is capable of inducing immune responses during leptospirosis. It can also strongly bind to laminin and collagen IV. We produced recombinant form of Lsa63 antigen and developed two ELISAs based on rLsa63 which were tested on human serum samples from patients with proven leptospirosis. These human serum samples were also examined by microscopic agglutination test (MAT) as a golden test for the diagnosis of Leptospirosis; the ELISA results were finally compared with the MAT results.

## 2. Objectives

To improve the previous works we developed an ELISA with novel recombinant leptospiral surface adhesion (Lsa63) protein to offer a new diagnostic test.

## 3. Material and Methods

### 3.1. Bacteria Strains

For performing MAT, a panel of Six *Leptospira interrogans* serovars including: grippotyphosa (ATCC 23469), pomona (ATCC 23478), icterohaemorrhagiae (ATCC 43642), canicola (ATCC 23606), hardjo (23480, L550) and serovar Ballum (serogroup Ballum, strain S 102) which are predominantly present in three northern provinces of Iran (Guilan, Mazandran and Golestan) (19) were maintained throughout the study.

### 3.2. Serum Samples

Sample size was calculated to be 154 according to following; in an experimental study; a total of 220 serum samples were obtained from 310 patients referred to *Leptospira* Central Laboratory of Guilan province for definitive diagnosis during March to August 2012. All 310 patients were suspected for leptospirosis according to WHO criteria including fever, myalgia, icterus, headache, stiff neck and also history of working in rice farm, to domestic or wild animals and surface contaminated water (14). Ninety serum samples were excluded from the study due to low volume (which must be at least 500 µL). All samples were tested in The Guilan province Central *Leptospira* Laboratory to confirm the diagnosis of leptospirosis (Equation 1). 

Equation 1. $$n=\frac{{\left[Z_{1}-\frac{\alpha }{2}(c+1)+Z_{1}-\beta \sqrt{{\left(c+1\right)}^{2}-{\left(c-1\right)}^{2}\times P}\right]}^{2}}{{\left(c-1\right)}^{2}\times P}={\frac{\left[1.96(3+1)+0.84\sqrt{{\left(3+1\right)}^{2}-{\left(3-1\right)}^{2}\times 0.22}\right]}{{\left(3-1\right)}^{2}\times 0.22}}^{2}=154$$

(Note: C = 3, P = 0.22, 1-α = 0.8 and 1-β = 0.95)

### 3.3. Microscopic Agglutination Test

The MAT was carried out according to standard methods (14, 20) using a panel of six reference *Leptospira interrogans* serovars mentioned above. Reciprocal agglutination titers of greater than or equal to 100 were considered as positive reactions.

### 3.4. Cloning and Recombinant Leptospiral Antigen Lsa63 Production

The reference *Leptospira* interrogans serovar Copenhageni genomic DNA was used for preparing rLsa63 antigen. The specific PCR primers utilized to amplify Lsa63 gene without signal peptide tag (1614 bp) were; Forward primer with an *NdeI* restriction endonuclease site (underlined): GCGCGGCAGCCATATGGAAAGTTCTAAACTCGGAG and reverse primer with an *XhoI* restriction endonuclease site (underlined): GGTGGTGGTGCTCGAGAATCAGTTTTAGATCGGC. Using Pfu polymerase, the *Lsa63* gene (LIC10314) was amplified (1614 bp) and then was cloned directly into an expression vector pET28a (+) at *NdeI* and *XhoI* restriction sites by Clontech cloning kit (Clontech, USA).

### 3.5. Expression and Purification

The recombinant plasmid [pET28a (+)-Lsa63] was transformed into *E. coli* BL21 (DE3) expression host cells. A single colony of the transformed cells [E.coli BL21(DE3)- pET28a (+)- Lsa63] was selected and grown overnight at 37˚C on 5 mL of Luria-Bertani (LB) Broth containing kanamycin to inoculate 500 mL of fresh LB broth containing kanamycin. The culture was grown with continuous shaking (250 rpm) at 37˚C to an optical density 0.6-0.8 at 600 nm and was induced by isopropyl-β-D-thiogalactoside (IPTG) at 1 mM final concentration. The cells were the allowed to grow for five more hours under constant agitation (250 rpm) at 20˚C. The culture pellet containing rLsa63 protein was harvested by centrifugation. The N-terminal His-tagged-fused Lsa63 protein was purified by Ni-NTA agarose (Qiagen USA) under the native condition. Finally, the first 2 mL of elution fractions which contained rLsa63 protein was collected. These fractions were pooled and dialyzed against phosphate buffered saline (PBS) pH = 7.4 to remove the imidazole. The efficiency of the rLsa63 purification was evaluated by 12% sodium dodecyl sulfate polyacrylamide gel electrophoresis (SDS-PAGE). The concentration of purified rLsa63 protein was measured by using Bradford method (21).

### 3.6. Western Blot Analysis

Following western blotting standard protocol the rLsa63 protein was analyzed using the anti-6His-tag antibody. Using the Bio-Rad system, resolved proteins were transferred to polyvinylidene fluoride (PVDF) membrane (20, 21). The blotting membrane was blocked for one hour with Tris-buffered saline (TBS) pH = 7.5 containing 5% (w/v) skim milk. The membranes were washed five times with TBS containing 0.05% Tween 20 (TBST) and then incubated for overnight in a proper dilution of peroxidase-conjugated mouse anti-His-tag at 4˚C. Finally, the membranes were washed five more times with TBST. Finally diaminobenzidine solution (DAB; sigma, USA) was added to paper to get the protein bands.

### 3.7. ELISA Establishing and Performance

Flat-bottom polystyrene microtiter plates (MaxiSorp, Nunc) were coated with 0.1 μg of purified rLsa63 antigen in pH 9.6 0.06 M carbonate-bicarbonate buffer overnight at 4˚C by checkerboard titration plates (6, 22, 23). Optimum concentration of the rLsa63 antigen, antibodies and conjugate were determined by checkerboard titrations plates (22). The plates were washed three times with TBST and then were incubated with 300 μL blocking solution (TBST with 2% w/v BSA) at 37˚C for one hour and finally was washed two times with TBST and dried. These coated plates were stored at -20˚C until use. Serum samples were diluted in TBST (1:50) then 50 μL of it was added to each well of ELlSA plates and incubated at 37˚C for 1 hour. Plates were washed five times with TBST. A 1:5000 dilution of rabbit anti-Human IgG HRP conjugate at 100 μL, was added to each well and incubated for 30 minutes at room temperature. Plates were washed five more times. Afterwards the TMB substrate solution was added to the wells at 100 μL and were left to react for 15 minutes at room temperature. When the color developed, 100 μL of stop solution (2 M H_2_SO_4_) was added to wells and the absorbance was measured using an ELISA reader at 450 nm. The mean cut-off point of OD450 + 3SD for Lsa63-ELISA was calculated twice by the mean value of OD from the test performed on 50 MAT negative control serum samples.

### 3.8. Statistical Analysis

Accuracy of the rLsa63-ELISA tests in human serum samples was evaluated by the sensitivity, specificity, positive predictive value (PPV), negative predictive value (NPV), positive likelihood ratio (PLR) and negative likelihood ratio (NLR) tests, with 95% confidence interval. All analyses were done using the user-written modules diagt in Stata software (release 10; StataCorp LP, College station, TX, USA), Youden’s index was also calculated (24).

## 4. Results

### 4.1. Serum Samples

Two hundred twenty human serum samples were collected. The mean and standard deviation of patients was 45 and 15, respectively. Other demographic information of patients has been summarized in Table 1. These sera were transferred under freezing condition to pathobiology department of Public Health Faculty of Tehran University of Medical Sciences and then stored at -20˚C until use.

**Table 1.** Demographic Characteristics of the Samples

Demographic Variable	No. (%)
**Sex**	
Male	128 (58.2)
Female	92 (41.8)
**Job**	
Farmer	211 (95.9)
Others	9 (4.1)
**Location areas**	
Rural areas	206 (93.6)
Urban areas	14 (6.4)

### 4.2. Cloning and Expression

**Figure 1.**
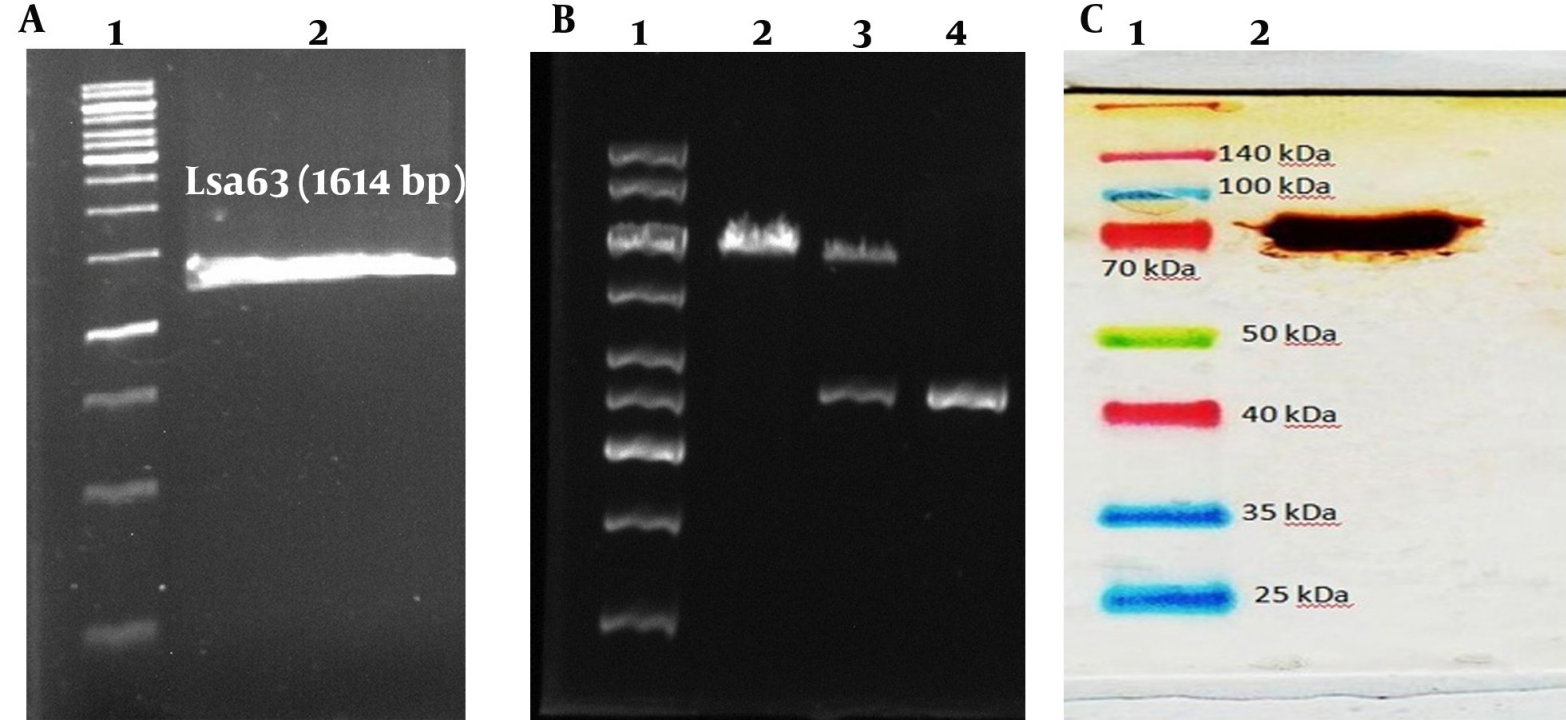
Lsa63 PCR Product, Double digestion of pET28 a(+)-Lsa63 and Western Blot of rLsa63 Protein A. lane 1, Kb DNA size marker; line 2, Lsa63-specific PCR products. B. Double digestion of pET28a (+)-Lsa63 using NdeI/XhoI: (left to right) line 1, 1 Kb ladder; line 2, recombinant plasmid; line 3, double digested product; line 4, PCR product of Lsa63. C. western blot of rLsa63 protein: (left to right) line 1, protein ladder; line 2, western blot of rLsa63.

The Lsa63 gene was amplified by Pfu polymerase enzyme. The agarose electrophoresis of Lsa63 PCR product showed an amplicon of Lsa63 at 1614 base pairs after ethidium bromide staining (Figure 1 A). The results of sequencing and double digestion of recombinant plasmid using NdeI and XhoI restriction enzymes demonstrated a successful cloning of Lsa63 gene into the pET28 a(+) (Figure 1 B). Lsa63 gene was expressed in *E. coli* (DE3). SDS-PAGE analysis of recombinant strains expressing lysate showed major identifiable band at about 70 kDa. The recombinant protein was purified and western blotting was performed using anti 6His-tag antibody (sigma. USA) (Figure 1 C). 

### 4.3. ELISA Establishing and Performance

The main difference between optical density of positive and negative sera in ELlSA was achieved by coating of 50 ng/well rLsa63 antigen, 1/50 dilution of serum and 1/5000 dilution of conjugate. The cutoff point from our 50 negative serum samples was 0.39. Among 220 serum samples, 30% (n = 65) were positive by MAT, and also 38% (n = 84) and 40.9% (n = 90) showed positive reaction by IgG and IgM rLsa63-ELISA, respectively. The rLsa63- ELISA tests had a 93.8% sensitivity (95% CI = 84.2-98.01), an 81.29 % specificity (95% CI = 74 -86.9) and a 85.0 (80.28-89.71) accuracy compared to MAT with titers ≥ 1:100 for IgM antibodies. Also, the test showed an 83.07% sensitivity (95% CI = 71.3-90.8), an 80.64% specificity (95% CI = 73.3- 86.3) and an 81.36 accuracy for IgG antibodies. The accuracy of the IgM ELISA and the IgG ELISA were 85.0 (95% CI = 80.28-89.71) and 81.36 (95% CI = 76.21-86.50), respectively. Youden’s indexes for IgM and IgG ELISA were calculated to be 0.75 and 0.63, respectively. Other statistical indexes have been summarized in Table 2. 

**Table 2.** The Performances of the Lsa63-ELISAs Tests, Compared to the MAT

Test Performances	IgM-Lsa63 ELISA	IgG-Lsa63 ELISA
**Positive predictive value, (%) (95% CI)**	67.77 (56.99-77.0)	64.28 (53.01-74.23)
**Negative predictive value, (%) (95% CI)**	96.9 (91.8-99.0)	91.91 (85.6-95.6)
**Positive likelihood ratio, (95% CI)**	5.01 (3.59-7.0)	4.29 (3.05-6.02 )
**Negative likelihood ratio, (95% CI)**	0.075 (0.029-0.196)	0.20 (0.122-0.360 )
**Test accuracy, (%) (95% CI)**	85.0 (80.28-89.71)	81.36 (76.21-86.50)

## 5. Discussion

The problem with leptospirosis is that its initial symptoms can easily be misdiagnosed as other infectious diseases (9). To overcome this predicament researchers have attempted many different approaches. The use of recombinant form of antigens as a diagnostic marker is safer than handling live Leptospires which is used in techniques such as MAT. Therefore, recombinant antigens can be more suitable for large scale serological examinations, routine diagnostic tests, epidemiology surveys and follow-up investigations of outbreaks. Many researchers have developed various methods with a number of recombinant *Leptospira* antigens such as LipL32, Lipl41, Lipl21, rLoa22 and Flagellin heat-shock protein to diagnose leptospirosis. Some information about these tests has been summarized in Table 3. In the present work, we performed further studies with Lsa63 protein as a new antigen introduced by Vieira ML and showed reactions with serum from patients with proven leptospirosis (13). This protein was expressed in native form. By using rLsa63 protein as a serological antigen, we developed IgM and IgG ELISAs and evaluated them for the serodiagnosis of human leptospirosis.

**Table 3.** Comparison of Different Tests Developed With Different Recombinant Antigens

Parameters Researchers	Recombinant Ag	Sensitivity	Specificity	Efficiency
**Senthilkumar, 2008**	rLipL41	83.33	93.07	87.78
**Dey et al. 2008 (6)**	LipL32	96.25	91.07	94.12
**Chalayon Piyanart, 2011**	Loa22	76.6	76.58	76.59
**Chalayon Piyart**	LipL32	56.6	88.7	84.6
**Chalayon Piyart**	rLipL41	70.0	74.6	74.0
**Joseph Siju, 2012 (12)**	rLipL21	100	97.09	97.5
**Our study-IgM, 2012**	Lsa63	93.8	81.29	85.0
**Our study-IgG, 2012**	Lsa63	83.7	80.64	81.36

Our results demonstrated that the sensitivity and specificity of Lsa63-ELISAs is good at detecting specific antibodies against *Leptospira* serovars. Our findings showed that the rLsa63 has a potential capability for improving the Leptospirosis diagnosis and can be used as a new antigen for developing new enzyme immunoassays for detecting anti *Leptospira* antibodies in the human sera. The sensitivity and specificity found in this study is comparable with one reported by Flannery et al. who evaluated a different ELISA with a different recombinant antigen for the diagnosis of human leptospirosis (25). It is noteworthy that less serum samples were used in that survey for evaluating the test. Dey et al. developed an ELISA with rLipL32 antigen for the detection of anti-leptospiral antibodies in human serum samples. The relative sensitivity, specificity and accuracy of ELISA developed in that study were higher than that of ours (6). Senthilkumar used rLipL41 antigen and established an ELISA for serodiagnosis of canine leptospirosis. The sensitivity and specificity of this ELISA were 83.33% and 93.07% respectively, similar to that of ours (22). Joseph Siju developed an ELISA with rLipL21 antigen to diagnose bovine leptospirosis. He determined the sensitivity of rLipL21 ELISA for 62 MAT positive cases to be 100% and the specificity with 378 MAT negative cases, 97.09%. Thus, he suggests that the rLipL21 protein-based ELISA could be used as an alternative to MAT for diagnosing of bovine leptospirosis (12). Chalayon Piyanart and et al. used LipL21, LipL32, LipL41 and Loa22 recombinant antigens to establish ELISA tests for serodiagnosing of leptospirosis. They obtained various range of sensitivity, specificity and accuracy for their tests. They reported that rLipL32 and rLoa22 gave efficiency of about 75% while the efficiency was about 62% for rLipL41 and 68% for rLipL21 (1).

To the best of our knowledge, this is the first prospective study of Lsa63 novel antigen used to establish an immunoassay for the diagnosis of leptospirosis. Our results showed an acceptable sensitivity, specificity and even Youden’s index for the detection of *Leptospira* infection in human. Youden’s index of 0.75 for IgM and 0.63 for IgG ELISA demonstrates a good agreement for these tests. However, validation of these tests in different populations is essential to be confirmed the present results. There is a need for further studies with greater numbers of subjects from different area to evaluate this method's accuracy in order to confirm it as as a practical diagnostic test for developing an ELISA kit for *Leptospira* infections. The results of our ELISA was obtained using a single sample (1/50 diluted serum). It is strongly suggested that future studies use two or more recombinant antigens with Lsa63. It is also worth mentioning that obtaining two serum samples from each one or two weeks apart could be of further help to validate the results. In conclusion, our findings suggest that Lsa63-ELISA is a specific, sensitive method and can be used as a practical test for the detection of antibodies against human leptospirosis.
